# Low-dose, high-pitch, spiral (FLASH) mode versus conventional sequential method for coronary artery calcium scoring: A derivation-validation study

**DOI:** 10.34172/jcvtr.31736

**Published:** 2024-03-13

**Authors:** Niraj Nirmal Pandey, Sayannika Chakraborty, Mansi Verma, Priya Jagia

**Affiliations:** Department of Cardiovascular Radiology & Endovascular Interventions, All India Institute of Medical Sciences, New Delhi, India

**Keywords:** Coronary artery disease, Coronary angiography, Cardiac-gated imaging techniques

## Abstract

**Introduction:**

The present study sought to compare the diagnostic accuracy and radiation dose of ECG-gated, ultra-fast, low-dose, high-pitch, spiral (FLASH) mode versus conventional, ECG-gated, sequential coronary artery calcium (CAC) scoring in patients with suspected coronary artery disease (CAD).

**Methods:**

The study included 120 patients who underwent both conventional scanning and FLASH mode scanning and were subdivided into derivation and validation cohorts. In the conventional sequential (step-and-shoot) protocol, prospective ECG-gated, non-contrast acquisition was performed at 70% of R-R interval. The spiral (FLASH) mode utilized a high-pitch and high-speed gantry rotation scanning mode where acquisition of the entire heart was done within a single cardiac cycle with prospective ECG-gating at 70% of R-R interval.

**Results:**

Correlation between CAC scores derived from conventional (cCAC) and FLASH mode (fCAC) in derivation cohort was excellent (r=0.99; *P*<0.001). A linear regression model was used to develop a formula for deriving the estimated CAC score (eCAC) from fCAC (eCAC=0.978 x fCAC). In validation cohort, eCAC showed excellent agreement with cCAC (ICC=0.9983; 95%CI: 0.9972 - 0.9990). Excellent agreement for risk classification (weighted kappa=0.93898; 95%CI: 0.86833 - 1.0000) was observed with 95% (57/60) scores falling within the same risk category. Effective dose was significantly lower in FLASH mode (conventional, 0.58±0.21 mSv vs. FLASH, 0.34±0.12 mSv; *P*<0.0001).

**Conclusion:**

CAC scoring using FLASH mode is feasible with high accuracy and shows excellent agreement with conventional CAC scores at significantly reduced radiation doses.

## Introduction

 Coronary artery calcification is a marker of atherosclerosis and the extent of coronary calcium can provide insight into the total burden of disease. There have been multiple studies suggesting that coronary artery calcification is an independent risk factor for major adverse cardiac events.^1,2^ Coronary artery calcium (CAC) scoring has role in risk prediction and prognostication in various populations and clinical situations.^3^ This method has been increasingly used to identify asymptomatic patients at high risk of major cardiac events so as to institute primary preventive therapies.^4^ ECG-gated sequential CAC scoring using computed tomography (CT) is the standard method for detection and quantification of coronary artery calcification.

 There has been pronounced interest in identifying low dose techniques for CAC scoring keeping in mind the ALARA (as low as reasonably achievable) principle.^5^ Multiple studies have compared various low-dose CT protocols with standard ECG-gated CAC scoring.^6,7^ Though low-dose CT examinations provided satisfactory results and risk stratification, the problems encountered with non-gated scans include increased image noise and motion artefacts resulting in low diagnostic accuracy. With the advent of dual source CT scanners with high-speed gantry rotation and high pitch scanning, the overall radiation burden can be reduced without affecting the overall image quality.^8-11^ While in the conventional sequential scanning, the data set is acquired over several heart beats, using the high pitch spiral scanning, the entire heart can be scanned within a single cardiac cycle.

 The present study sought to investigate the performance of ECG-gated ultra-fast, low-dose, high pitch, spiral (FLASH) mode versus the conventional ECG-gated sequential CAC scoring with regards to the diagnostic accuracy and radiation dose in patients with suspected coronary artery disease (CAD).

## Materials and Methods

###  Patient selection

 This was a prospective study conducted at a tertiary medical center. Ethical approval was obtained from the Institutional Ethics Committee and written informed consent was obtained from all patients. All consecutive patients aged 35 or above with suspected CAD who referred for CT angiography from June, 2021 to August, 2021 were recruited. The exclusion criteria included any known history of coronary stent implantation or coronary artery bypass graft surgery that might compromise the assessment of CAC, scans with non-diagnostic image quality and patients who did not provide informed consent. A total of 125 patients were screened for the study of which five patients were excluded due to previous history of coronary artery bypass graft surgery (n = 3) and coronary stent placement (n = 2), resulting in a final study population of 120 patients.

 The study population was divided into two groups; the first 60 patients to be recruited were included in the derivation cohort and the latter 60 recruited patients served as the validation cohort. The demographic information (i.e., age, gender and heart rate) and scan parameters (i.e., scan duration, tube voltage and pitch) were recorded for all patients.

###  CT acquisition protocol 

**Figure 1:**
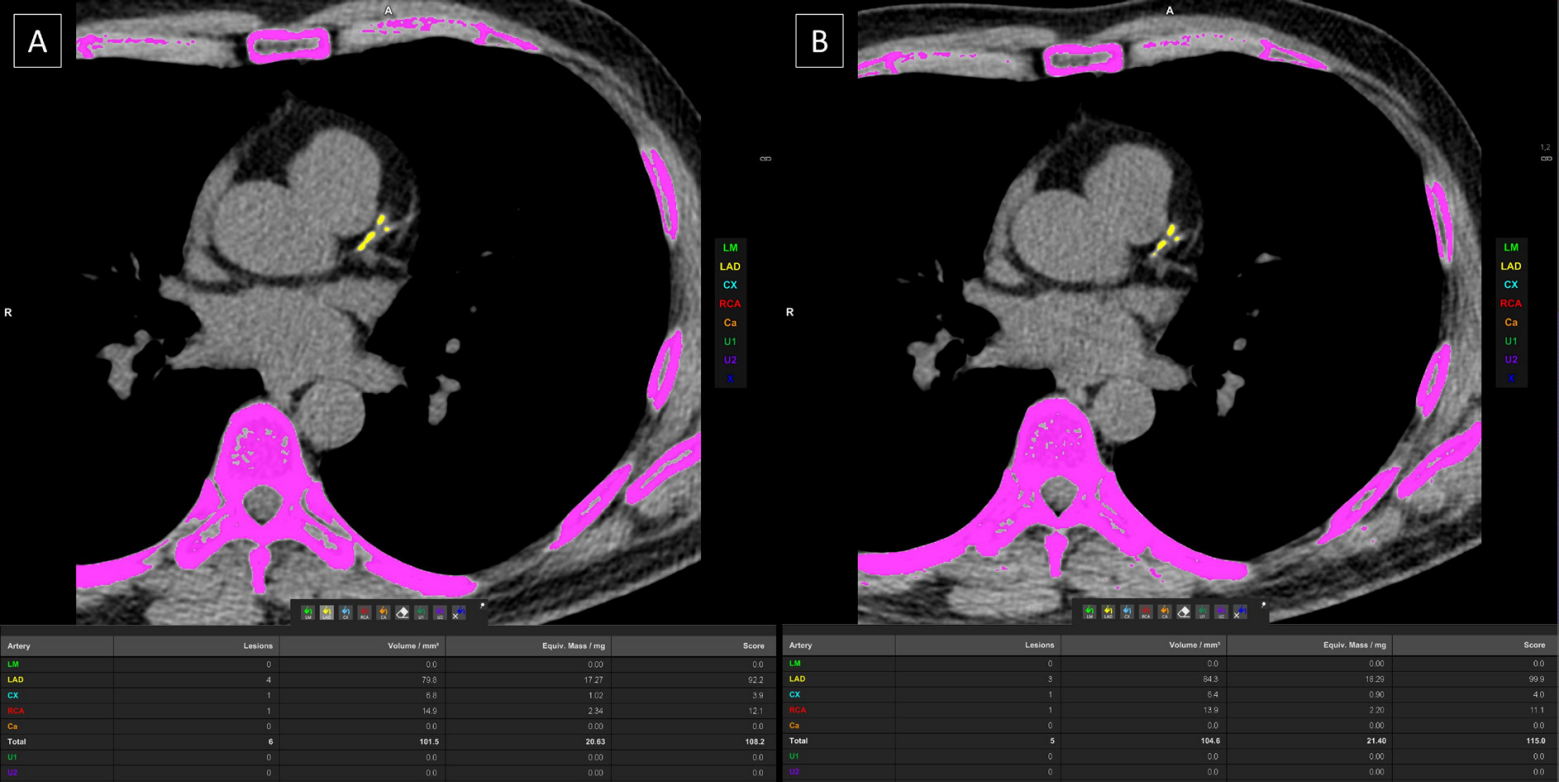
Coronary artery calcium (CAC) scoring using conventional sequential protocol **(A)** and the spiral (FLASH) mode protocol **(B)** showing the CAC scores to be 108.2 and 115 respectively

 All examinations were performed on a 384-slice third generation dual source CT scanner, SOMATOM FORCE (Siemens Healthineers, Forchheim, Germany) with temporal resolution of 66 milliseconds. CAC scoring was performed using both the conventional sequential protocol and the spiral (FLASH) mode protocol. Images were obtained extending from the carina to the cardiac apex. In the conventional sequential (step-and-shoot) protocol, prospective ECG-gated, non-contrast acquisition was performed at 70% of R-R interval. The imaging parameters included: tube voltage-120 kV, automated tube current modulation (CARE Dose4D, Siemens Healthcare, Forchheim, Germany) with reference tube current being 80 mAs. The images were reconstructed in the mediastinal window with slice thickness of 3 mm and increment of 1.5 mm using Qr36 kernel with model-based iterative reconstruction strength level 3 (ADMIRE; Siemens Healthcare, Forchheim, Germany) (Figure 1A).

 The spiral (FLASH) mode utilized a high-pitch and high-speed gantry rotation scanning mode where acquisition of the entire heart was done within a single cardiac cycle with prospective ECG-gating at 70% of R-R interval. The imaging parameters included: tube voltage: 120 kV, pitch: 3.2 and automated tube current modulation (CARE Dose4D, Siemens Healthcare, Forchheim, Germany). Slices were reconstructed in mediastinal window of 3.0 mm section and increment of 1.5 mm using Qr36 kernel with model-based iterative reconstruction strength level 3 (ADMIRE; Siemens Healthcare, Forchheim, Germany) (Figure 1B).

###  Image analysis

 The Agatson score was calculated for both scans using dedicated software (CaScoring, Syngo.via, Siemens, Erlangen, Germany). The software predefines the area with attenuation value > 130 HU and the user can select the calcification related to coronary arteries. The Agatston method utilizes the weighted sum of the selected “lesions” (the area of calcification is multiplied by a factor determined by the maximum lesion attenuation – plaques having attenuation 130-199 HU, 200-299 HU, 300-399 HU, and ≥ 400 HU are assigned factors 1, 2, 3 and 4 respectively. The data evaluation was performed by two experienced cardiac radiologists with at least 5 years of experience in reporting of cardiac CT images. Based on the Agatston scores, risk stratification was evaluated on the scale of 0-3, where 0 (CAC score = 0) depicted low-risk category, 1 (CAC score between 0.1 – 99.9) depicted intermediate-risk category, 2 (CAC score between 100 - 399.9) depicted high-risk category and 3 (CAC score ≥ 400) depicted very high-risk category of patients. CT dose index volume (CTDI_vol_) and dose-length product (DLP) were considered as comparative dose measurements for both the scanning protocols.

###  Statistical analysis

 The data was summarized and analyzed using MedCalc Statistical Software version 18.2.1 (MedCalc Software bvba, Ostend, Belgium; http://www.medcalc.org; 2018). Continuous variables were expressed as mean values ± standard deviations and were compared using t-test. Categorical variables were depicted as frequencies or percentages and compared using chi square or Fischer exact test as appropriate. A linear regression model was used in the derivation cohort to obtain correction factor and derive a formula which was subsequently tested in the validation cohort. The inter-technique agreement for continuous variables was assessed using intraclass correlation coefficient and Bland-Altman plot and using weighted kappa for ordinal variables. A p-value of 0.05 or less was considered as statistically significant.

## Results

 The study population comprised of 120 patients (mean age: 51.78 ± 9.45 years [Range: 38-76 years]; 78/120 (65%) males) who met the inclusion and exclusion criteria. The mean heart rate at acquisition was 67.37 ± 7.67 beats per minute (Range: 48-83 beats per minute). All patients underwent scanning using both protocols, viz. routine sequential ECG-gated mode followed by high pitch FLASH mode, for CAC score assessment. The 120 patients were divided into two cohorts, viz. derivation and validation cohort. The initial 60 patients comprised the derivation cohort (mean age: 49.90 ± 9.19 years [Range: 38-76 years]; 42/60 (70%) males). The following 60 patients (mean age: 53.67 ± 9.41 years [Range: 39-75 years]; 36/60 (60%) males) comprised the validation cohort.

 The mean Agatston score on routine conventional ECG-gated scan (cCAC) and FLASH mode scan (fCAC) was 112.8 ± 379.16 and 118.35 ± 399.34 respectively. Excellent agreement was found between cCAC and fCAC scores (ICC = 0.9969; 95% CI: 0.9955 - 0.9978). Of the 120 patients, a positive ( > 0) CAC score was observed in 49 (40.83%) patients using the conventional method and 51 (42.5%) patients using the FLASH mode. Considering the values obtained using the conventional method as the gold standard, 3 false positive results and 1 false negative result was observed using the FLASH mode. In the 3 patients with false positive results, the Agatston score using FLASH mode was 0.3, 1.2 and 4.2 respectively while in the patient with a false negative result, the Agatston score using the conventional method was 0.3.

###  Derivation cohort

**Figure 2:**
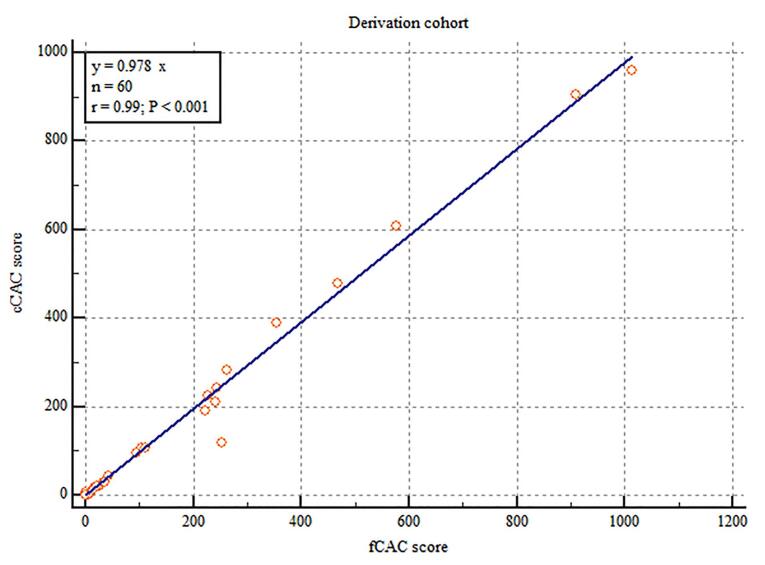
Correlation between coronary artery calcium scores obtained from conventional method (cCAC score) and FLASH mode (fCAC score) in the derivation cohort

**Figure 3:**
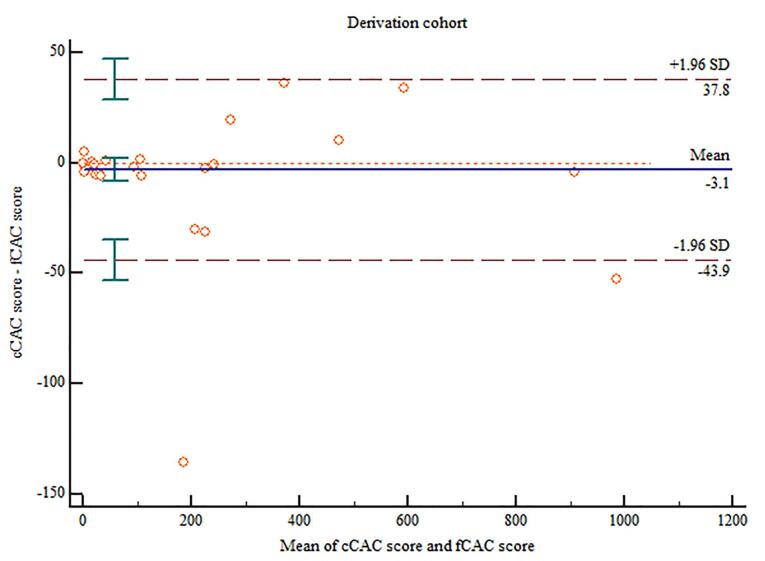
Bland-Altman plot comparing the coronary artery calcium scores obtained from conventional method (cCAC score) and FLASH mode (fCAC score) in the derivation cohort

 The cCAC and fCAC score was 84.91 ± 200.79 and 88 ± 203.81 respectively. Linear regression model depicted excellent correlation between cCAC and fCAC scores (r = 0.99; *P* < 0.001) (Figure 2). A multiplication factor was calculated with the help of the linear regression data keeping the y-intercept as 0 and a slope was obtained which gave the ratio between cCAC and fCAC scores (Estimated calcium score [eCAC] = 0.978 x fCAC). Excellent agreement was found between cCAC and fCAC scores in this cohort (ICC = 0.9947; 95% CI: 0.9911 - 0.9968) and no significant proportional bias was observed using the Bland-Altman plot (Figure 3).

###  Validation cohort

**Figure 4:**
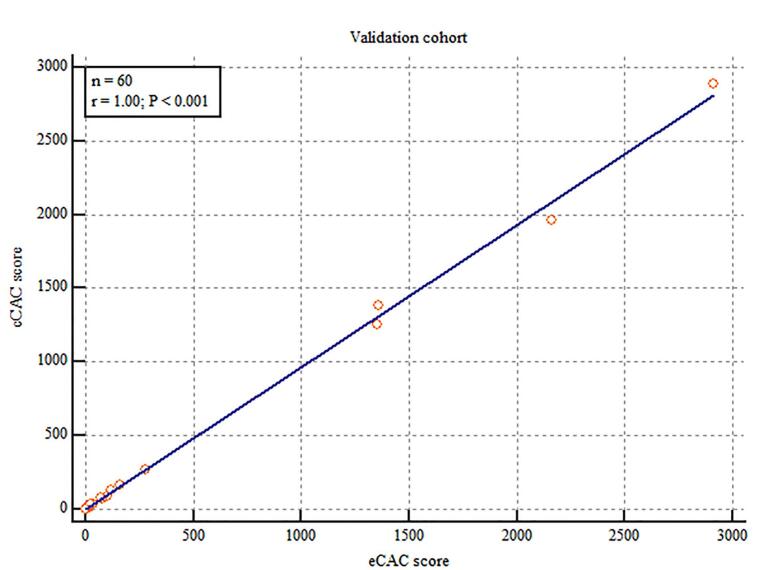
Correlation between coronary artery calcium scores obtained from conventional method (cCAC score) and estimated score (eCAC score) derived from the FLASH mode in the validation cohort calculated by the formula eCAC = 0.978 x fCAC. [fCAC: coronary artery calcium scores obtained from FLASH mode]

**Figure 5:**
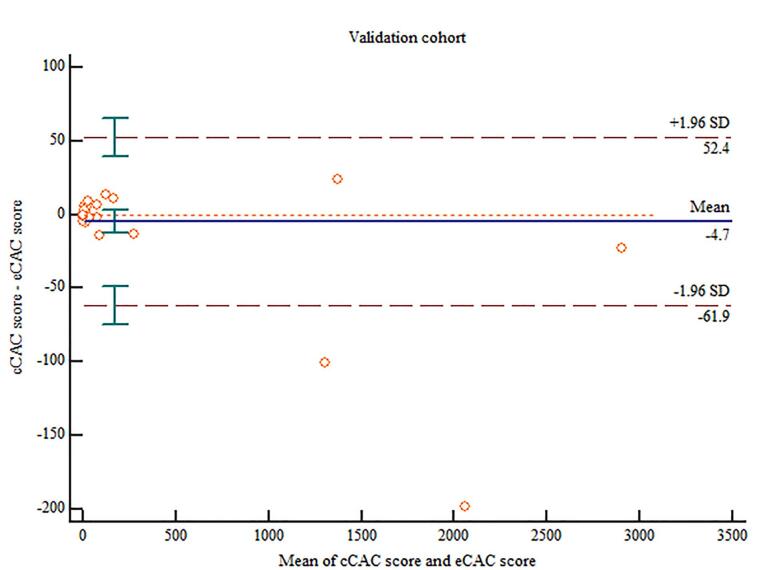
Bland-Altman plot comparing the coronary artery calcium scores obtained from conventional method (cCAC score) and the estimated score (eCAC score) derived from the FLASH mode in the validation cohortcalculated by the formula eCAC = 0.978 x fCAC. [fCAC: coronary artery calcium scores obtained from FLASH mode]

 The cCAC and fCAC score was 140.72 ± 498.05 and 148.7 ± 527.48 respectively. Using the equation derived from the linear regression in derivation cohort (eCAC = 0.978 x fCAC), the estimated coronary artery calcium score was calculated (mean eCAC: 145 ± 515.87). Linear regression model depicted excellent correlation between cCAC and eCAC scores (r = 1.00; *P* < 0.001) (Figure 4). Excellent agreement was found between cCAC and eCAC in this cohort (ICC = 0.9983; 95% CI: 0.9972 - 0.9990) and no significant proportional bias was observed using the Bland-Altman plot (Figure 5).

###  Reclassification of risk

 The reclassification in risk stratification in the validation cohort is depicted in Table 1. Using the eCAC scores derived from the FLASH mode, reclassification occurred for 2 (3.33%) patients from low- to intermediate-risk category, and for 1 (1.67%) patient from intermediate- to low-risk category. No reclassification of risk was observed in patients in the high- and very high-risk category. Excellent agreement for risk classification (weighted kappa = 0.93898; 95%CI: 0.86833 – 1.0000) was observed between cCAC and eCAC scores.

**Table 1:** Reclassification of risk according to calcium scores in the validation cohort

**eCAC score**	**cCAC score**				
	**0.0**	**0.1-99.9 **	**100-399.9**	**≥400**	
0.0	33	1	0	0	34 (56.67%)
0.1-99.9	2	17	0	0	19 (31.67%)
100-399.9	0	0	3	0	3 (5%)
≥ 400	0	0	0	4	4 (6.67%)
	35 (58.33%)	18 (30%)	3 (5%)	4 (6.67%)	

cCAC: coronary artery calcium scores obtained using conventional method; eCAC: estimated score derived from the FLASH mode in the validation cohort calculated by the formula, eCAC = 0.978 x fCAC [fCAC: coronary artery calcium scores obtained from FLASH mode]

###  Radiation dose assessment

 Themean CT volume dose index and DLP was 2.84 ± 1.15 mGy and 41.26 ± 15.14 mGy.cm respectively for the conventional method and 1.35 ± 0.49 mGy and 24.6 ± 8.48 mGy.cm respectively for the FLASH mode. Using a conversion factor (*k =*0.014 mSvmGy^-1^ cm^-1^), the mean effective dose was 0.58 ± 0.21 mSv for the conventional method compared to 0.34 ± 0.12 mSv for FLASH mode (*P* < 0.0001), resulting in a 40.48% reduction in radiation dose while using the FLASH mode.

## Discussion

 The present study has highlighted the advantage of using a high-pitch, spiral (FLASH) mode acquisition over conventional mode acquisition for quantification of coronary artery calcium and risk stratification. Using the FLASH mode, excellent diagnostic accuracy can be obtained at reduced radiation doses with optimal risk stratification.

###  Agatston score evaluation and risk stratification

 In the present study, the validation of the Agatston score datasets with the help of linear regression model resulted in a more concrete conclusion that the CAC scores obtained in FLASH mode were in excellent agreement with those obtained using the conventional method. Furthermore, the FLASH mode had relatively higher sensitivity for calcium detection and was able to detect calcifications in 3 patients where the conventional method showed a ‘zero’ CAC score.

 In a previous study by Hutt A et al, it was observed that a non-gated, high-pitch scan could be used to obtain reliable detection as well as quantification of CAC.^12^ However, some contradictory CAC scores were observed which were attributed to motion artifacts on account of using a non-gated scan protocol.^12^ In another study by Shin et al, CAC scores were overestimated in the non-gated scan.^13^ This problem was obviated in the present study where prospective ECG-gating was used in the high-pitch scan protocol.

 The difference in calcium scores between the two scanning methods depended linearly on each other, i.e., increased with increasing calcium scores which is similar to what has been observed in previous studies.^7,14^ In the present study, no significant difference was observed in the risk stratification using the eCAC scores derived from the FLASH mode in the validation cohort. This is especially important as no detectable calcification is associated with very low risk of developing cardiovascular disease.^15^ In the present study, reclassification occurred for 2 patients from low- to intermediate-risk category, and for 1 patient from intermediate- to low-risk category. The reclassification rate in our study remains within the expected limits as reclassification up to 2-9% has been reported by other studies.^16,17^

###  Radiation dose reduction

 Previous studies have determined that while technical advancements and advanced dose reduction techniques have resulted in significant decrease in the radiation burden incurred in CT coronary angiography, there has been little change in the radiation dose incurred in a CAC scoring scan as the conventional method still utilizes sequential scanning at 120 kV. The results of our study revealed that using an ECG-gated FLASH mode, a radiation dose reduction of approximately 40% was possible without compromising the diagnostic accuracy. The results are concordant with the study by Vonder et al where significant reduction in radiation dose (48%) was obtained using the high-pitch mode.^16^

 We acknowledge the following limitations. This was a single center study with a small sample size. The ultra-fast, low-dose, high-pitch, spiral (FLASH) mode acquisition can only be performed in latest generations of dual-source CT scanner. The patient population was not strictly homogenous, as all patients with suspicion of CAD referred for CT angiography were included irrespective of the pain characterization or the pre-test probability of CAD.

## Conclusion

 The current study has derived and validated a formula that enables CAC scoring using ultra-fast, low-dose, high-pitch, spiral (FLASH) mode acquisition with high accuracy and shows excellent agreement with the conventional CAC scores at a significantly reduced radiation dose. It can potentially be implemented in routine clinical practice for risk stratification in patients with suspicion of CAD without any compromise in the diagnostic accuracy while significantly reducing the radiation-related risks.

## Competing Interests

 The author(s) declared no potential conflicts of interest with respect to the research, authorship, and/or publication of this article.

## Ethical Approval

 The study was approved by the Institutional Ethics Committee at All India institute of Medical Sciences, New Delhi (IECPG-235/24.03.2021, RT-43/28.04.2021). Written and informed consent was obtained from all the participants.

## Funding

 Authors received no financial support for the research, authorship, and/or publication of this article.
